# Multimode fibre: Light-sheet microscopy at the tip of a needle

**DOI:** 10.1038/srep18050

**Published:** 2015-12-14

**Authors:** Martin Plöschner, Věra Kollárová, Zbyněk Dostál, Jonathan Nylk, Thomas Barton-Owen, David E. K. Ferrier, Radim Chmelík, Kishan Dholakia, Tomáš Čižmár

**Affiliations:** 1 Ewing Building, School of Engineering, Physics & Mathematics, University of Dundee, Dundee, DD1 4HN, Scotland; 2 SUPA, School of Physics and Astronomy, University of St Andrews, St Andrews, Fife, KY16 9SS, Scotland; 3 CEITEC - Central European Institute of Technology, Brno University of Technology, Technická 10, Brno 616 00, Czech Republic; 4 The Scottish Oceans Institute, Gatty Marine Laboratory, University of St Andrews, St Andrews, Fife, KY16 8LB Scotland; 5 School of Medicine, University of St Andrews, St Andrews, Fife, KY16 9TF Scotland

## Abstract

Light-sheet fluorescence microscopy has emerged as a powerful platform for 3-D volumetric imaging in the life sciences. Here, we introduce an important step towards its use deep inside biological tissue. Our new technique, based on digital holography, enables delivery of the light-sheet through a multimode optical fibre – an optical element with extremely small footprint, yet permitting complex control of light transport processes within. We show that this approach supports some of the most advanced methods in light-sheet microscopy: by taking advantage of the cylindrical symmetry of the fibre, we facilitate the wavefront engineering methods for generation of both Bessel and structured Bessel beam plane illumination. Finally, we assess the quality of imaging on a sample of fluorescent beads fixed in agarose gel and we conclude with a proof-of-principle imaging of a biological sample, namely the regenerating operculum prongs of *Spirobranchus lamarcki*.

Development of methods for fast, high-resolution volumetric imaging of living specimens has been central to numerous great advancements across several disciplines of life sciences, particularly developmental biology and neuroscience^1^. Light-sheet (LS) fluorescence microscopy has already proven itself to be a particularly eligible choice as it allows for fast, high-contrast sectioning of the specimen with a minimum level of phototoxicity from sample irradiance^2^,^3^,^4^. With the immense success of this new technique in recent years, we see a burgeoning need for its application deeper into living tissues; to enable observation of important cellular processes within their natural environment – the complexity of the living organism. When penetrating deeper into living specimens, the LS experiences rapid degradation of its wavefront due to the highly scattering nature of tissues^5^. The first strategy to increase penetration depth has been the employment of multi-photon excitation since scattering at longer wavelengths is much weaker. This has enabled imaging at depths up to ≈1000 *μ*m^6^,^7^,^8^. Separately, methods based on beam-shaping have been introduced that enable either generation of LS shapes much less sensitive to scattering^9^,^10^ or direct wavefront correction of the LS using adaptive optics^11^,^12^. The last emerging technology aims to introduce LS illumination via minimally invasive endoscopic devices. A recently demonstrated endoscope^13^ delivers the LS illumination via a gradient-index lens thus reducing the footprint of the instrument below 1 mm.

In this paper we introduce a new approach, providing the LS delivery via an extremely narrow multimode fibre (MMF). MMFs have been only recently identified as reliable optical elements capable of both the beam delivery^14^,^15^,^16^ and scanner-free imaging^17^,^18^. This allows for utilisation of MMF as a miniature microscope objective for LS purposes. We use holographic wavefront engineering to pre-shape the coherent laser signals coupled to the proximal end of the MMF in order to obtain the LS at the distal end of the instrument. This concept requires no anterior optics, therefore the total instrument footprint can be as small as the fibre core (a few 10 s of *μ*m).

In addition, MMFs feature a remarkably faithful cylindrical symmetry that can be very efficiently exploited to enhance the LS performance. Due to the diffraction of light, the most commonly used Gaussian beam (GB) LS profiles are associated with an unavoidable trade-off between the axial resolution and the size of the field of view^19^,^20^. However, by utilising the cylindrical symmetry of MMFs, which prevents coupling of light between mode-groups having different propagation constants^21^, we can readily synthesise a pseudo-nondiffracting Bessel beam (BB)^17^. BB LS enables an extended uniform illumination of the specimen^10^ and thus a very significant extension of the field of view. The undesired contribution of the concentric rings^9^ can be virtually eliminated by complementing the BB LS with the methods of structured-illumination (SI)^22^,^23^. This significantly improves the axial resolution and contrast.

In the following, we first describe the wavefront shaping methods for the generation of all the above types of the LSs at the end of MMFs. We then progress to a detailed analysis of the performance and imaging quality of all these LS modalities using a model sample of fluorescent beads fixed in agarose gel. Finally, we conclude with a proof-of-concept demonstration – imaging the regenerating operculum of *Spirobranchus lamarcki* using all of the available LS modalities.

## Results

### Generation of light-sheet at the multimode fibre output

The generation of a LS at the end of a MMF requires the knowledge of its full transformation matrix 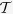, which unambiguously relates the input and output fields. For this purpose, we adopt the 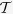 matrix acquisition approach described in^24^.

**Figure 1:**
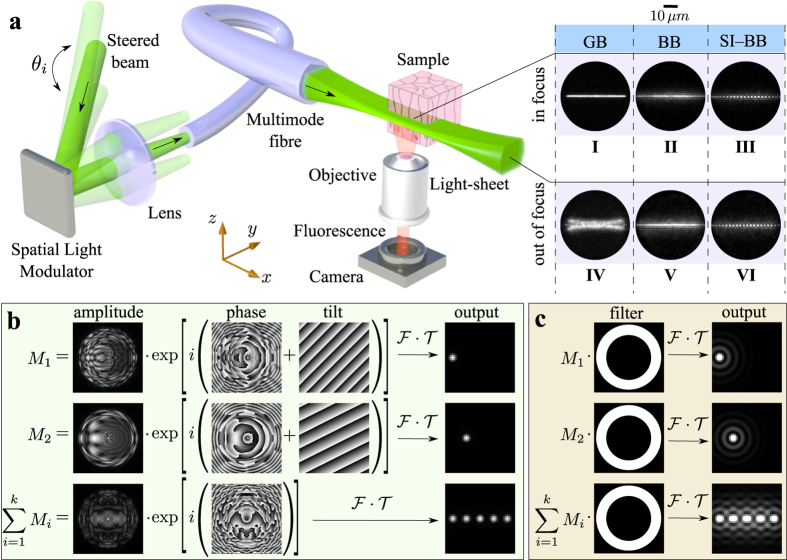
Light-sheet pattern generation. (**a**) A simplified scheme of the LS delivery using the MMF (detailed experimental setup is in supplementary information S4). The insets (I–VI) show the *yz*-plane profiles of the GB, BB and SI-BB LS in focus (*x* = 50 *μ*m from fibre facet; LS scanning shown in Media. 1–3) and out of focus (*x* = 100 *μ*m from fibre facet; LS scanning shown in Media. 4–6). (**b**) A strategy for obtaining the SLM hologram generating the GB LS at the fibre output. (**c**) A procedure converting the GB LS to a BB/SI-BB LS.

The principle of LS formation behind a MMF (see Fig. 1a) is similar to the digital scanned light-sheet microscopy (DSLM)^3^. A laser beam (Verdi V5, *λ* = 532 nm) illuminates an SLM (BNS 512 × 512) that is programmed to display a single mask, which consists of a sum optical fields 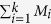 (Fig. 1b). The holographic mask 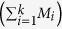 contains all the information required to generate a LS line at one particular vertical (*z*) position in the calibration plane. Each complex field *M*_*i*_ is designed in a way such that – after passing through a lens (Fourier transform 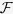) and a fibre (transformation matrix 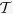) – it creates a single focal point in a certain position of the fibre output plane (calibration plane). Each of the *M*_*i*_ fields is complemented by a unique tilt grating that changes the direction of its propagation off axis by an angle *θ*_*i*_. Steering the SLM illumination with an acousto-optic deflector (AOD), we can redirect each of the *M*_*i*_ fields into the fibre sequentially and at very high refresh rates. Choosing the positions of all the resulting focal points along a single line in the calibration plane, the full scanning cycle of AOD delivers a complete LS pattern that is used for fluorescent sample excitation. In order to enable 3-D imaging, we design a series of SLM holographic masks 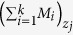 where 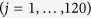, that each produce the LS line at different vertical (*z*_*j*_) positions in the calibration plane. Displaying all the focal points sequentially, in time discrete intervals, not only prevents the undesired interference effect between neighboring points^24^, but it also brings all the advantages of the DSLM approach: the adjustable scanning range, uniform LS power density and the ability to introduce structured illumination^3^,^25^.

In this proof-of-concept study, where we focus on the LS delivery, a microscope objective lens is used to orthogonally collect the fluorescence emission and form an image of the illuminated plane on a CCD detector. A more advanced future form of this method will use a separate MMF to image the sample plane onto a detector. The exposure time of the CCD is set to an integer multiple of AOD duty cycles. As seen in other DSLM approaches, the integration of the signal during the scanning of the point decreases the signal to background ratio of the method due to additive background noise. This is an intrinsic feature of DSLM-like methods. The axial position of the detection objective is controlled by a piezo stage ensuring that the detection focal plane overlaps with the position of the LS at all times during the 3-D imaging.

The generation of the advanced forms of LS is based on converting the GB focal points into a BB (Fig. 1c). Conveniently, this is possible even without elaborate beam-shaping approaches due to the cylindrical symmetry of the fibre. The symmetry leads to conservation of propagation constants *k*_*z*_ of the MMF’s eigenmodes^17^,^21^,^26^. The BBs, which have an extremely narrow *k*_*z*_ spectra, can therefore be formed by restricting the *k*_*z*_ components of the light entering the MMF – simply by activating only a thin annular zone of the SLM hologram. Consequently, we can use the same holographic modulations *M*_*i*_ as in the case of GB LS. In this case, however, the modulations are multiplied by a binary filter with a thin annular aperture. In order to optimise the parameters of the annular filter, we have studied this behaviour numerically using a simulated transformation matrix. The parametric study of the resulting BB characteristics – the central core size and the axial extent is summarised in supplementary information S1, S2.

Generating BB-lattices for structured-illumination BB (SI-BB) LS follows essentially the same procedure as in the BB LS modality. The individual cores of BBs, however, do not overlap, but require rather carefully optimised spacing^22^ for efficient numerical suppression of the side lobes.

### Comparison of light-sheet profiles

The 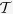 matrix can be acquired at any distance beyond the fibre facet. In the following, we measured the 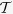 in a *yz*-plane at a distance of *x* = 50 *μ*m from the fibre facet. This allows non-contact imaging of the fluorescent sample at the maximum numerical aperture (NA = 0.22) of the fibre^17^, theoretically enabling 1.2 *μ*m spot size for a GB at this plane. The quality of the LS patterns is validated with an auxiliary camera. The insets (I–VI) of Fig. 1a show the obtained verification profiles of the generated GB, BB and one lattice of SI-BB LSs in the plane of calibration (in focus; *x* = 50 *μ*m from fibre facet) and also at the distance of *x* = 100 *μ*m from the fibre facet (out-of-focus). The comparison of (I) and (IV) profiles clearly demonstrates the diverging nature of the GB LS resulting in a reasonable axial resolution only in the close proximity of the focus. In contrast, BB LS (II,V) and SI-BB LS (III,VI) are basically independent of the distance from the calibration plane, which is highly desirable for imaging of extended specimens.

**Figure 2:**
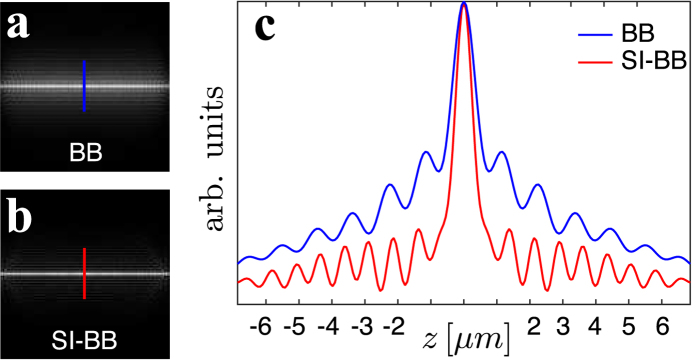
Comparison of the BB/SI-BB LS approach. The simulated intensity profile of (**a**) BB LS and (**b**) SI-BB LS in the calibration (focal) plane. (**c**) One dimensional intensity profile taken from (**a**) (blue line) and (**b**) (red line) respectively.

In order to compare the axial resolution of the BB/SI-BB LS, we have numerically calculated their profiles at the end of the fibre (Figs 2a,b). The BB LS (Fig. 2a) profile suffers from concentric rings of BB cores, which will lead to the excitation of out-of-plane regions and, as a result, deteriorate the optical sectioning power. The SI-BB LS profile is emulated by creating a series of *N* = 6 horizontally displaced BB lattices with the image intensity *I*_*n*_. Adding up the lattices using 
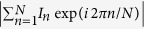
 results in a SI-BB LS profile that is almost free of the outer ring structure (Fig. 2b). The same numerical approach is applied to analysis of fluorescent images acquired by this method. As the SI-BB LS requires *N* = 6 frames at each LS plane, the acquisition of the sample volume data is six times slower than in the case of the BB and GB LS. The level of suppression of the outer ring structure with respect to the central core intensity is clearly visible on a comparison between the BB and the SI-BB LS profiles (Fig. 2c). The efficiency of removing the outer rings strongly depends on the selected lattice constant and on the size of the BB core^22^.

### Imaging of fluorescent particles: resolution studies

**Figure 3:**
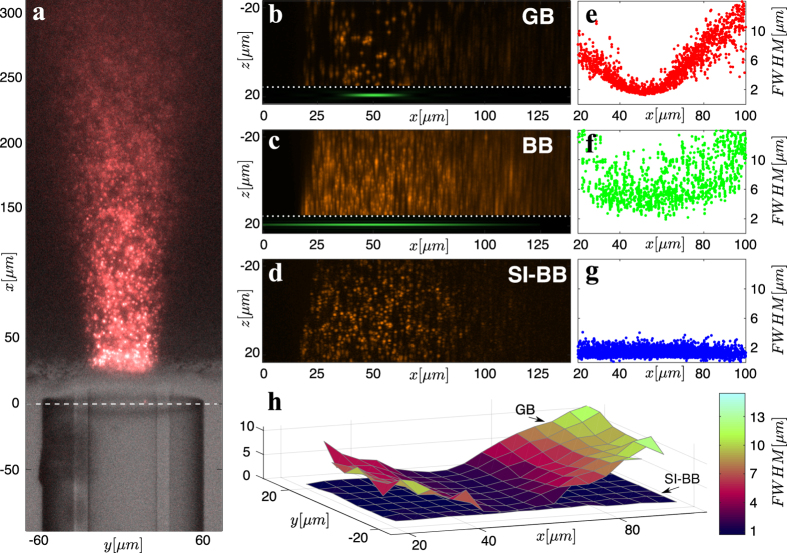
Axial resolution studies. (**a**) An image from the detection objective with the control illumination turned on. The MMF is at the bottom while the agarose gel with dispersed fluorescent particles is at the top. The control view is superposed with GB LS fluorescence frame. The maximum intensity projection along the *y*-axis for the (**b**) GB LS (Media. 7), (**c**) BB LS (Media. 8) and (**d**) SI-BB LS (Media. 9). The insets in (**b**,**c**) show the axial extent of GB and BB/SI-BB beam. We do not show the inset in (**d**) as it is equivalent to (**c**). The measured FWHM of 1 *μ*m fluorescent particles for the case of the (**e**) GB LS, (**f**) BB LS and (**g**) SI-BB LS. (**h**) The comparison of the FWHM *xy*-map for the GB and SI-BB LS.

A multimode fibre (Thorlabs FG050UGA) with a 50 *μ*m core and 125 *μ*m cladding diameter respectively, is used to deliver the excitation light to the vicinity of a sample – the agarose gel with dispersed fluorescent particles (red fluorescent dye FireFli Red; 1 *μ*m diameter) (Fig. 3a). The agarose gel droplet is cut by a blade, which creates a flat wall allowing simple fibre access. The droplet is suspended on a standard glass cover slide mounted on a xyz-micrometer stage. The sample is moved into close vicinity of the fibre and the excitation LS pattern is scanned by the SLM in a vertical fashion and in steps of 0.4 *μ*m. The fibre and the sample remain fixed during this process while the piezo z-stage moves the detection objective in perfect synchronisation with the light-sheet motion to ensure that the fluorescent emission images stay in focus. The whole stack acquired consists of 120 slices.

The imaging quality of all modalities is compared in Fig. 3b–d using the maximum intensity projection method^27^ for volume visualisation. The GB LS (Fig. 3b, Media. 7) provides satisfactory axial resolution only in the vicinity of the calibration plane. Similarly to previous studies^9^, the BB LS (Fig. 3c, Media. 8) provides stable axial resolution in the observed field of view but its axial resolution suffers due to the presence of the rings surrounding the central core. The SI-BB LS (Fig. 3d, Media. 9) provides constant and superior axial resolution up to about *x* = 100 *μ*m from the fibre facet. Here, the intensity starts to decrease due to the limited axial extent of the BB used (see inset of Fig. 3c).

The three-dimensional stacks shown in Fig. 3b–d were acquired at various locations of the agarose gel in order to build up robust statistical information on the axial resolution as a function of the distance from the fibre facet for all three LS modalities. The results are shown in Fig. 3e–g. Figure 3e clearly shows that the best axial resolution of roughly 1.8 *μ*m, determined as the full width at half maximum (FWHM), is achieved at the distance of approximately 50 *μ*m (the calibration plane) for the GB LS. Performing the imaging any closer or further away from the fibre facet leads to a deterioration of the axial resolution. The axial resolution of BB LS (Fig. 3f) has the average value of only 9 *μ*m due to out-of-plane excitation. The SI-BB LS (Fig. 3g) provides a stable and superior axial resolution of approximately 1.2 *μ*m up to a distance of at least 100 *μ*m from the fibre facet.

Figure 3h shows the axial resolution as a function of both *x* and *y* for the case of GB and SI-BB LSs. There is a significant variation of the axial resolution in the field of view for a GB LS; on the other hand, the axial resolution of a SI-BB LS is fairly uniform over the whole field of view.

### Imaging of Spirobranchus lamarcki

**Figure 4:**
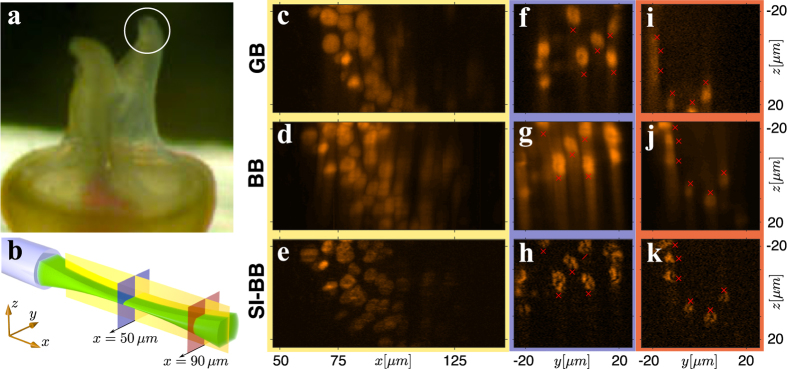
Imaging of biological sample. (**a**) Close-up image of operculum prongs of *S. lamarcki*. (**b**) The cross-sections used for the comparison of the imaging performance of GB, BB and SI-BB LS modalities. (**c**,**f**,**i**) GB LS, (**d**,**g**,**j**) BB LS and (**e**,**h**,**k**) SI-BB LS. (**c**–**e**) are accompanied with Media. (10–12) showing rotation of the sample around the *x*-axis. A small sample drift was present between the imaging of GB and BB/SI-BB modalities as indicated by the red markers.

We have demonstrated the quality of the MMF LS microscope on a sample of *Spirobranchus lamarcki* that is regenerating its operculum. The preparation of the sample is described in supplementary info S3. We have imaged the prong-like protrusions on the end of the operculum (Fig. 4a), a structure used by *S. lamarcki* to protect the opening of its habitation tube. These regenerating *S. lamarcki* opercula offer a new system in which to study animal appendage regeneration and biomineralisation^28^.

We compare the performance of GB, BB and SI-BB LS modalities in three relevant cross-section planes (Fig. 4b). The yellow cross-section reveals the imaging performance over an extended field of view, whereas the blue and red cross-sections compare the LS modalities in focus and out-of-focus respectively. Figure 4c–e (yellow cross-section) show that the GB LS (Fig. 4c, Media. 10) provides good axial resolution only in a limited field of view. The BB LS (Fig. 4d, Media. 11) offers constant but inferior axial resolution due to the out-of-focus fluorescence excitation. The SI-BB LS (Fig. 4e, Media. 12) clearly provides the best performance and enables solid axial resolution up to a distance of *x* = 100 *μ*m from the fibre facet, where the BB core intensity starts to fade. Figure 4f–h show the blue cross-section through another sample that had some fluorescent nuclei in the calibration plane (50 *μ*m). The SI-BB cross-section (Fig. 4h) provides the best image quality and reveals clearer detail of the structure in the nuclei that is also present in (Fig. 4f,g), but barely recognisable. Finally, Fig. 4i–k show the red cross-section through the sample at the distance of *x* = 90 *μ*m. The GB LS axial resolution, at this distance from the fibre facet, fails to resolve three nuclei to the left of the image (Fig. 4i) that are otherwise clearly visible in the SI-BB case (Fig. 4k).

## Discussion

In this paper we have demonstrated a novel route for light-sheet microscopy, where the excitation signal is holographically shaped and delivered into the sample via an extremely narrow multimode optical fibre (proximately 125 *μ*m in diameter). Its cylindrical symmetry facilitates implementation of advanced light-sheet modalities based on the generation of a Bessel beam light-sheet complemented with structured illumination – a combination allowing for a significant increase in the resolution and the accessible field of view.

Testing this approach by imaging fluorescent spheres, we have reached close to isotropic axial resolution of 1.2 *μ*m in a significantly extended volume – about an order of magnitude larger when compared to results obtained with the standard Gaussian light-sheet profile. Furthermore, we have not observed any significant vibrations affecting the fibre-tip in our experiments, which holds great promise for future *in vivo* applications. The reason behind this exceptional stability of the fibre tip stems from the fact that the fibre is not scanned during volumetric image acquisition, but instead, the light-sheet is scanned by means of holographic beam-shaping. The imaging of the biological sample showed exceptional imaging quality in a cluster of cells, thus demonstrating the suitability of the technique for biological applications.

The current experimental geometry could already be advantageous in many practical applications (e.g. on-chip integration), however, in-depth *in-vivo* light-sheet microscopy is yet to come. This requires complementing the excitation pathway with a separate scanner-free collection geometry (e.g.^17^,^18^) and optimising its efficiency on broadband spectra^29^. We foresee that a wholly fibre based light-sheet microscope would take the form of two parallel optical fibres (or a single dual-core fibre) with carefully designed termination to maintain the orthogonal orientation of excitation and collection pathways. In addition, there is a potential to reduce the acquisition time by several orders of magnitude by using alternative spatial light modulators based on digital micromirror devices^23^.

*In vivo* implementation of the technology will very likely result in fibre bending, which affects the transformation matrix and this, in turn, affects the quality of the light-sheet. The ability to correct for such changes on-the-fly and without the access to the distal end of the fibre is therefore highly desirable. Significant progress in this area has been reported recently, based on the accurate numerical prediction of the transformation matrix of the bent fibre without the need for its empirical measurement^30^. Previously, fibre bending required repeated experimental measurement of the transformation matrix, which is relatively time consuming even when employing the latest and fastest technology^15^. The new approach reduces the problem to the determination of the fibre conformation, which can be, at least in principle, performed quickly with three detectors of moderate resolution but with large penetration depth. Furthermore, the new approach also potentially enables numerical determination of transformation matrices for multiple wavelengths, which would enable multicolor excitation experiments at the distal end of the fibre.

We believe there are no fundamental obstacles in realisation of the described microendoscopic device, which will truly revolutionise the field of light-sheet microscopy and enable, for the first time, minimally-invasive deep tissue light-sheet microscopy. Future research efforts will be aimed at delivering the light-sheet through fibres based on soft glasses. Such fibres allow for numerical apertures equivalent to that of high numerical aperture microscope objectives.

## Additional Information

**How to cite this article**: Plöschner, M. *et al*. Multimode fibre: Light-sheet microscopy at the tip of a needle. *Sci. Rep*. **5**, 18050; doi: 10.1038/srep18050 (2015).

## Supplementary Material

Supplementary Information

Supplementary Movie S1

Supplementary Movie S2

Supplementary Movie S3

Supplementary Movie S4

Supplementary Movie S5

Supplementary Movie S6

Supplementary Movie S7

Supplementary Movie S8

Supplementary Movie S9

Supplementary Movie S10

Supplementary Movie S11

Supplementary Movie S12

Supplementary Movie S13

Supplementary Movie S14
