# Supplementary material for: Multimode fibre: Light-sheet microscopy at the tip of a needle
Source: Sci Rep. 2015 Dec 14;5:18050. doi: 10.1038/srep18050 (PMC4677299; doi:10.1038/srep18050)
Supplement: Supplementary Information [file srep18050-s1.pdf]

Supplementary Information - Multimode fibre: Light-sheet microscopy at the tip of a needle

Martin Plöschner[1,2], Věra Kollárová[3], Zbyněk Dostál[3], Jonathan Nylk[2], Thomas Barton-Owen[4], David E. K. Ferrier[4], Radim Chmelík[3], Kishan Dholakia[2], and Tomáš Čižmár[1,5,*]

[1] *Ewing Building, School of Engineering, Physics & Mathematics, University of Dundee, Dundee, DD1 4HN, Scotland*
[2] *SUPA, School of Physics and Astronomy, University of St Andrews, St Andrews, Fife, KY16 9SS, Scotland*
[3] *CEITEC - Central European Institute of Technology, Brno University of Technology, Technická 10, Brno 616 00, Czech Republic*
[4] *The Scottish Oceans Institute, Gatty Marine Laboratory, University of St Andrews, St Andrews, Fife, KY16 8LB Scotland*
[5] *School of Medicine, University of St Andrews, St Andrews, Fife, KY16 9TF Scotland*
[*] t.cizmar@dundee.ac.uk

S1 Creating the bessel beam at the end of multimode fibre

The point sources lying on a circle of radius R_{SLM} in the SLM plane all generate plane waves with the same k_z (axial component of the k_0 vector) in the SLM's Fourier plane (fibre input plane). Since the k_z component of the field launched into a fibre is conserved as it propagates through to the other end, we have a simple way to generate Bessel beams at the fibre output. In practice, we do not select a circle of radius R_{SLM}, but rather an annular section of width d. Both the radius R_{SLM} and the width d of the annular section at the SLM influence critical parameters of the Bessel beam for imaging applications – the core radius r_c and the axial length l_c over which the Bessel beam core exists. As the choice of the optimal parameters differs depending on the sample imaged, we have developed a theoretical model that predicts these beam parameters and enables precise engineering and informed design of the Bessel beam at the fibre output.

First, we need to find the eigenmodes of the fibre. We adopt the scalar mode approach that provides sufficiently accurate values of the eigenmode profiles and eigenmode propagation constants $\beta_{l,m}$ (l is the topological charge and m is the radial index of the mode) for our application purposes. The more elaborate vector mode approach introduces very small corrections to propagation constants and, crucially, almost negligible corrections to the eigenmode profiles that are used for beam shaping at the fibre output facet. As a result, the Bessel beam parameters will be virtually unaffected by the choice of the scalar mode approach. The eigenmodes of the fibre can be written as:

$$\psi_{l,m} = F_{lm}(R)\exp(il\varphi) \qquad \dots \quad \beta_{l,m} \tag{S1}$$

where $R = r/a$ is a dimensionless radial parameter with r being the radial coordinate and a the radius of the fibre core; φ is the azimuthal angle. $F_{lm}(R)$ is the radial profile of the mode defined by Bessel functions of the first (J_l) and second kind (K_l):

$$F_{lm}(R) = \begin{cases} \frac{J_l(uR)}{J_l(u)}, \text{for } R < 1 \\ \frac{K_l(uR)}{K_l(u)}, \text{for } R \geq 1. \end{cases} \tag{S2}$$

Parameter u is proportional to the transverse wavevector of a considered mode, $u = a\sqrt{k_0^2 n_{\text{core}}^2 - \beta^2}$, where $k_0 = \omega/c$ is the vacuum wavenumber and n_{core} is the refractive index of the fibre core. The allowed parameters of u for a given fibre geometry are found by solving the following characteristic equation:

$$u\frac{J_{l-1}(u)}{J_l(u)} + \sqrt{v^2 - u^2}\frac{K_{l-1}(\sqrt{v^2 - u^2})}{K_l(\sqrt{v^2 - u^2})} = 0, \tag{S3}$$

where $v = ak_0\text{NA}$. NA is the numerical aperture of the fibre defined as $\text{NA} = \sqrt{n_{core}^2 - n_{clad}^2}$, with n_{clad} being the refractive index of the fibre cladding. Eq. S3 generally results in multiple solutions for each topological charge l. These solutions are indexed with the radial parameter m.

The parameters of the fibre used in the experiment are: core radius $a = 25\,\mu$m; numerical aperture NA = 0.217; refractive index of the core $n_{core} = 1.4666$. At the wavelength of $\lambda = 532\,nm$ this results in 1035 modes supported by the fibre. One such mode is shown on Fig. S1.

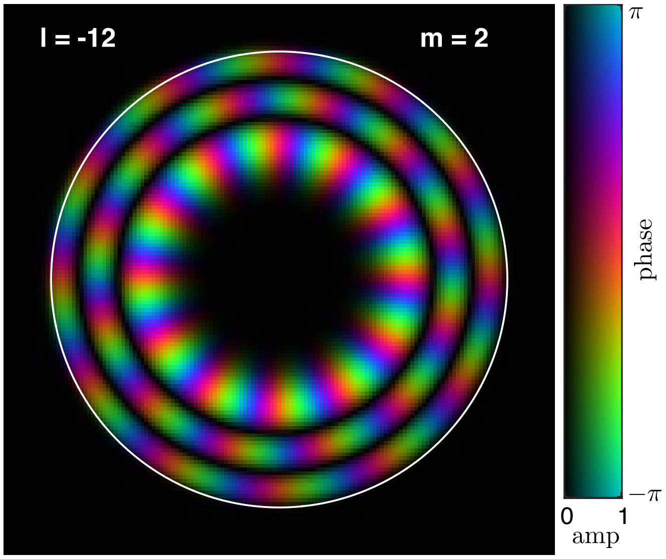

Figure S1. One of the modes supported by the fibre. All the modes supported are shown in Media. S1.

At this point, we divide the output of the fibre into a grid $(x \times y)$ of $N \times N$ points. We chose $N = 145$ throughout this section. The grid can contain a circle of $R = 1.2$, which ensures plenty of space to encode the exponentially decaying cladding profiles. We want to be able to display a point at any position within this grid. To be able to do that, we need to find the coefficients of the linear combination of the modes creating each individual point of the output grid – we need to solve the linear system of equations $\mathbf{AX} = \mathbf{B}$. Each column of matrix $\mathbf{A}$ (size of $\mathbf{A}$ is $(N^2 \times 1035)$) represents one particular eigenmode in a vector form $v^{l,m}$ of size $(N^2 \times 1)$. The required output matrix $\mathbf{B}$ is simply an identity matrix of size $(N^2 \times N^2)$. After solving this linear system of equations, each column of $\mathbf{X}$ (size $1035 \times N^2$) contains the coefficients for generation of one particular point at the fibre output. One point generated in this way is shown at Fig. S2a. The solution automatically finds the smallest possible spot that can be generated from a given set of fibre eigenmodes.

We can back-propagate the output field F_{out} creating this spot to the input facet of the fibre by multiplying each mode with $\exp\left(i\beta_{l,m}z\right)$. We back-propagate the field through a fibre that is $z = 1$ m long. As the propagation constants differ in between the individual modes, the input field F_{in} resembles a speckle pattern (Fig. S2b). F_{in} is the field we need to create on the SLM in order to get a point at the fibre output. In order to find the representation of the field on the SLM, we first rescale F_{in} (we use Fourier type rescaling to avoid introducing additional spacial frequencies) to a grid of 49×49 pixels (59×59 with part of the cladding), which is the setting of our experimental system. We then Fourier transform the field onto SLM (Fig. S2c). Here, we multiply the field with a binary filter represented by an annular section of central radius $R_{\text{SLM}} = 212$ and with the width of $d = 45$ pixels (Fig. S2d). We inverse Fourier transform the field back into the fibre input plane and rescale the field back to its 145×145 size (Fig. S2e). Now we find the coefficients of the eigenmodes generating this field by solving a linear system of equations, where $\mathbf{B}$ is now the annually filtered input field. We then propagate the field to the fibre output plane, which results in a point in the same position (x, y) as the original point but with the distinctive rings around it (Fig. S2f). This is the process by which the Bessel beam is created at the output facet of the fibre.

In the experimental geometry, the transformation matrix provides relation between input fields F_{in} and their respective output points. The F_{in} is of course created on the SLM. Multiplying the SLM mask with a binary filter with a thin annular section therefore produces the Bessel beam at the fibre output without any additional effort.

S2 The axial extent and the core size of the Bessel beam

In order to achieve optimal imaging results in the structured Bessel beam LS, the spacing between the beam cores and the size of the beam cores themselves has to be carefully chosen in order to generate a strongly modulated beam profile for the cores and a weakly modulated intensity profile for the outer rings. There is also an additional parameter that is mutually intertwined with the Bessel beam core size – the axial extent of the Bessel beam. The discrete grid of positions available for the output

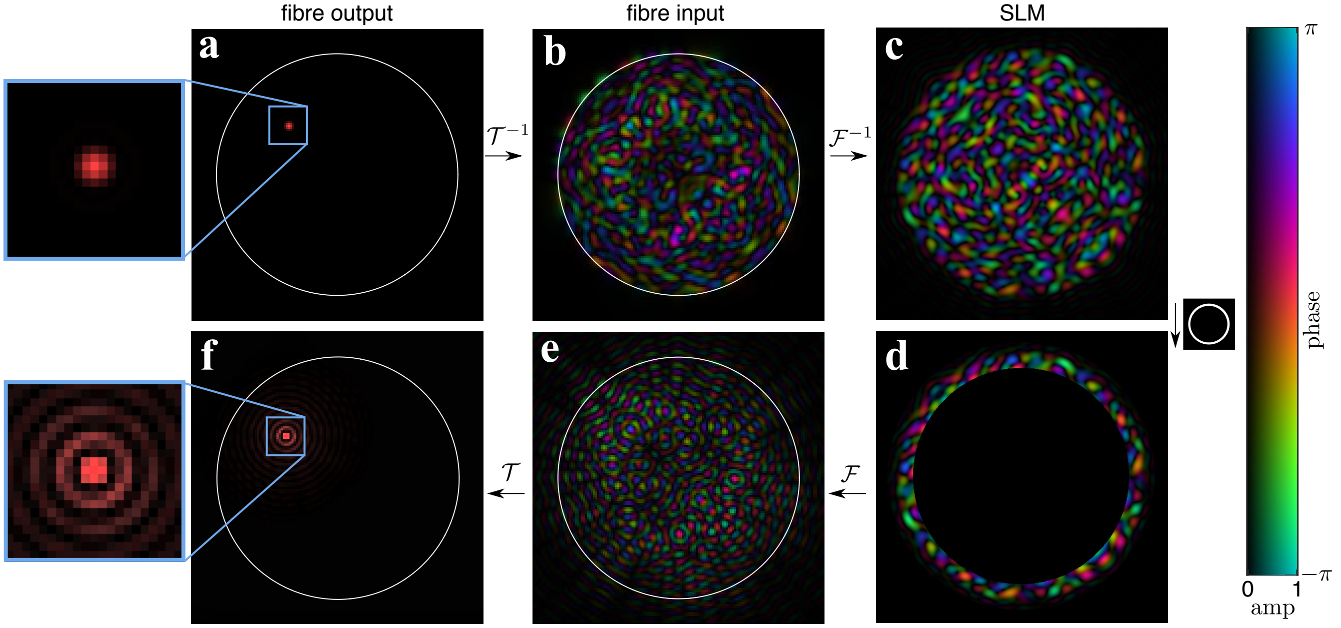

Figure S2. Generation of Bessel beam at the output facet of the fibre. (**a**) Point at the fibre output. (**b**) Field at the fibre input facet that generates the point at the output as in (**a**). (**c**) Representation of input field (**b**) at the SLM. (**d**) SLM field (**c**) multiplied by a binary filter with a thin annular section. (**e**) Field at the input facet of the fibre after application of the annular filter on the SLM. (**f**) The final point at the fibre output. The simulation for all the positions of output points is shown in Media. S2.

points of the fibre also restricts the optimal parameter space. A parametric study is therefore needed to engineer the lattice of optimal quality.

Figs. S3a-c, show the axial extent, the core size and the efficiency of the generation of a Bessel beam for various annular sections on the SLM. Fig. S3a shows that the axial extent of the Bessel beam at the output of the fibre is the longest (550 μm) for annular section of small radius k_r and small annular width Δk_r. Unfortunately, the core size is also very large at around (3 μm) (Fig. S3b). This makes perfect sense: the larger the beam the smaller the diffraction as it propagates and vice versa. The comparison of Fig. S3a and Fig. S3b also shows that the smallest core and acceptable axial extent is achieved for large k_r and very small Δk_r – again an expected behaviour. The last thing to consider is the efficiency of the Bessel beam generation (Fig. S3c). Obviously, a very thin annular section uses only a small fraction of the light coming to the SLM and it is therefore not very practical to choose infinitely small Δk_r – especially when considering relatively weakly fluorescing biological samples.

In light of these simulations and also considering the restraints for optimal removal of the outer ring structure, we have selected the $k_r = 2\,\mu\text{m}^{-1}$ and $\Delta k_r = 0.4\,\mu\text{m}^{-1}$ in our experimental geometry of BB and SI-BB LS. This choice leads to a reasonable axial extent of 120 μm and the core size (FWHM) of 0.8 μm, which is in good agreement with our experimentally observed results. The GB LS uses the whole area of the SLM and enables FWHM of 1.2 μm in the focus of the GB beam.

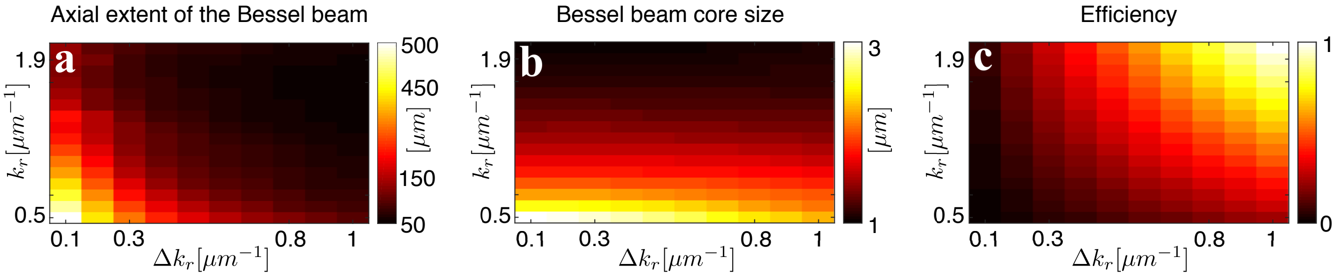

Figure S3. The parametric study of (**a**) the axial extent, (**b**) the core size and (**c**) the generation efficiency of the Bessel beam as a function of the reciprocal space radius of annular section k_r and its width Δk_r.

S3 Preparation of *Spirobranchus lamarcki*

Spirobranchus (formerly Pomatoceros) lamarcki specimen collection, husbandry and preparation was carried out according to the protocol described in Szabó and Ferrier.[1] After 6 days of regeneration, opercula were severed at or near the plane of the easy break point and were fixed in 4% (w/v) PFA in MOPS buffer (0.5 M NaCl, 2 mM MgSO 4, 1 mM EGTA, 0.1 M morpholinopropanesulfonic acid buffer, pH 7.5) for 1 h at room temperature. They were then washed 3 times in PBT (1$\times$ phosphate buffered saline solution, 0.1% Triton X-100) before being permeabilized in PBT for 1 hour at room temperature. The specimens were washed in 2x SSC (0.3 M NaCl, 0.03 M sodium citrate, pH 7.0) before being treated with 100 μg/mL DNase-free RNase A (Promega) in 2x SSC at 37 $^{\circ}C$ for 20 minutes. This was followed by four washes of 2xSSC. The samples were then stained using a 1:1650 (v/v) dilution of propidium iodide (PI, Life Technologies) in 2xSSC for 15 minutes at room temperature. Samples were washed twice in 2xSSC and then returned to PBT for transport, storage and imaging.

S4 Experimental setup

The beam delivery path used for coupling the light into both the SMF and MMF is depicted in Fig. S4a. The principle of volumetric imaging of the sample is shown in Fig. S4b. The sample is not present in Fig. S4b during the empirical measurement of the transformation matrix.

References

1. Szabó, R. & Ferrier, D. E. K. Cell proliferation dynamics in regeneration of the operculum head appendage in the annelid Pomatoceros lamarckii. *Journal of Experimental Zoology Part B: Molecular and Developmental Evolution* **322**, 257–268 (2014).

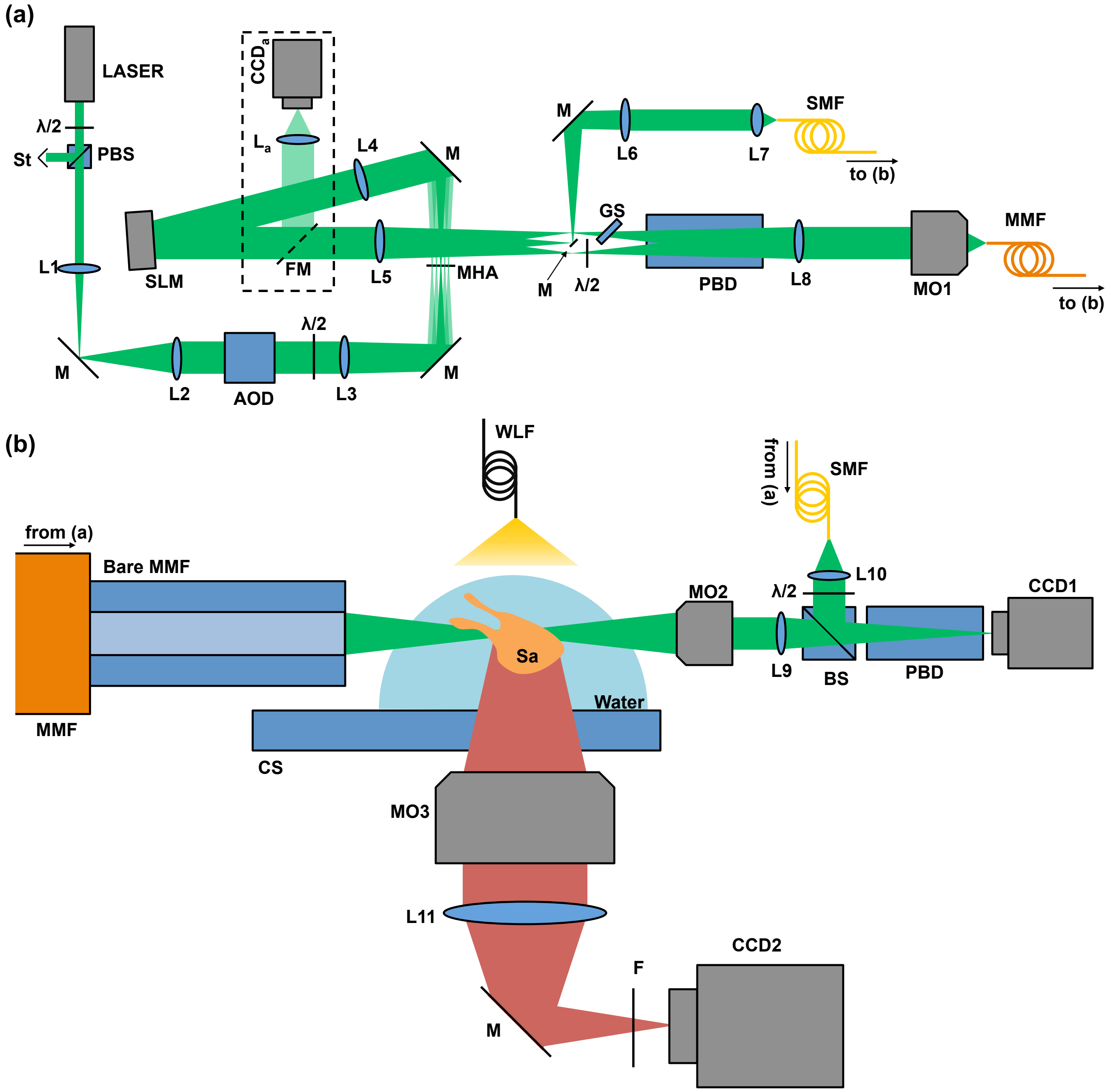

Figure S4. (**a**) Beam-delivery part of the setup; (**b**) (no sample present) T-matrix measurement part of the setup; (sample present) light-sheet scanning part of the setup. Focal lengths of achromatic doublet lenses L1-11 are f1 = 50mm, f2 = 200mm, f3 = 150mm, f4 = 200mm, f5 = 300mm, f6 = 150mm, f7 = 25.4mm, f8 = 400mm, f9 = 200mm, f10 = 25.4mm, f11 = 100mm, fa = 100mm, MO1 = Olympus Plan N 10x/0.25 Air (inf/-/FN22), MO2 = Mitutoyu 20x/0.42 Air, MO3 = Newport 20x/0.4 Air, St=Stop, PBS = Polarising beam splitter, AOD = Acousto-optic deflector, MHA = Micro-hole array, SLM = Spatial light modulator, FM = Flip mirror, GS = Glass slide, PBD = Polarising beam displacer, MOx = Microscope objective (xth), SMF = Single-mode fibre, MMF = Multi-mode fibre, WLF = White-light fibre, BS = beam splitter (50:50), F = Filter, CS = Coverslip, Sa = Sample.
